# Challenges in using transcriptome data to study the c-di-GMP signaling network in *Pseudomonas aeruginosa* clinical isolates

**DOI:** 10.1093/femsmc/xtad012

**Published:** 2023-07-18

**Authors:** Melisa Gür, Jelena Erdmann, Anke Will, Ziwei Liang, Jens Bo Andersen, Tim Tolker-Nielsen, Susanne Häussler

**Affiliations:** Institute for Molecular Bacteriology, TWINCORE, Centre for Experimental and Clinical Infection Research, Feodor-Lynen-Strasse 7, 30265 Hannover, Germany; Institute for Molecular Bacteriology, TWINCORE, Centre for Experimental and Clinical Infection Research, Feodor-Lynen-Strasse 7, 30265 Hannover, Germany; Institute for Molecular Bacteriology, TWINCORE, Centre for Experimental and Clinical Infection Research, Feodor-Lynen-Strasse 7, 30265 Hannover, Germany; Costerton Biofilm Center, Department of Immunology and Microbiology, Faculty of Health and Medical Sciences, University of Copenhagen, Blegdamsvej 3B 24.1, 2100 Copenhagen, Denmark; Costerton Biofilm Center, Department of Immunology and Microbiology, Faculty of Health and Medical Sciences, University of Copenhagen, Blegdamsvej 3B 24.1, 2100 Copenhagen, Denmark; Costerton Biofilm Center, Department of Immunology and Microbiology, Faculty of Health and Medical Sciences, University of Copenhagen, Blegdamsvej 3B 24.1, 2100 Copenhagen, Denmark; Institute for Molecular Bacteriology, TWINCORE, Centre for Experimental and Clinical Infection Research, Feodor-Lynen-Strasse 7, 30265 Hannover, Germany; Department of Molecular Bacteriology, Helmholtz Centre for Infection Research, Inhoffenstrasse 7, 38124 Braunschweig, Germany; Department of Clinical Microbiology, Copenhagen University Hospital – Rigshospitalet, Ole Maaloes Vej 26, 2100 Copenhagen, Denmark

**Keywords:** *Pseudomonas aeruginosa*, clinical isolates, alginate, c-di-GMP, transcriptional regulation, diguanylate cyclase

## Abstract

In the *Pseudomonas aeruginosa* type strain PA14, 40 genes are known to encode for diguanylate cyclases (DGCs) and/or phosphodiesterases (PDEs), which modulate the intracellular pool of the nucleotide second messenger c-di-GMP. While in general, high levels of c-di-GMP drive the switch from highly motile phenotypes towards a sessile lifestyle, the different c-di-GMP modulating enzymes are responsible for smaller and in parts nonoverlapping phenotypes. In this study, we sought to utilize previously recorded *P. aeruginosa* gene expression datasets on 414 clinical isolates to uncover transcriptional changes as a result of a high expression of genes encoding DGCs. This approach might provide a unique opportunity to bypass the problem that for many c-di-GMP modulating enzymes it is not known under which conditions their expression is activated. However, while we demonstrate that the selection of subgroups of clinical isolates with high versus low expression of sigma factor encoding genes served the identification of their downstream regulons, we were unable to confirm the predicted DGC regulons, because the high c-di-GMP associated phenotypes were rapidly lost in the clinical isolates,. Further studies are needed to determine the specific mechanisms underlying the loss of cyclase activity upon prolonged cultivation of clinical *P. aeruginosa* isolates.

## Teaser

We aimed for exploiting previously recorded transcriptional profiles of clinical isolates in order to define sets of genes whose expression is influenced by the activity diverse c-di-GMP modulating enzymes.

## Introduction

The ubiquitous second messenger bis-(3'5')-cyclic di-GMP (cdiGMP) regulates the transition between motile, planktonic and sessile, biofilm-associated lifestyles in a wide range of bacteria (Jenal and Malone 2006, Hengge 2009). The intracellular level of cdiGMP is tightly controlled by the opposing activities of two classes of enzymes, diguanylate cyclases (DGCs) and phosphodiesterases (PDEs). In many cases, the active GGDEF and EAL/HDGYP domains of DGCs and PDEs respectively are fused to transmembrane or other signal input domains thereby integrating numerous environmental and cellular stimuli into the cdiGMP signaling network (Galperin and Nikolskaya 2007, Ryan et al. 2012, Sondermann et al. 2012, Chua et al. 2017, Galperin and Schultz 2019). The group of Hengge (2009) has shown that the c-di-GMP modulating enzymes exhibit surprisingly distinct and specific output functions (Sarenko et al. 2017). To explain the specificity, they proposed ‘local’ c-di-GMP signalling, which involves direct interactions between specific DGC/PDE pairs and c-di-GMP-binding effector/target systems. Thus, while the totality of c-di-GMP-modulating enzymes generally drives the global switch from a highly motile and virulent bacterial phenotype to a sessile lifestyle within biofilm structures, the various players and subsets of c-di-GMP-modulating enzymes appear to be responsible for smaller and partially nonoverlapping bacterial phenotypes (Kulesekara et al. 2006; D. G. Lee et al. 2006).

*Pseudomonas aeruginosa* is an opportunistic pathogen that can cause severe acute as well as difficult to treat chronic biofilm-associated infections. The genome of the *P. aeruginosa* type strain PA14 encodes for overall 40 c-di-GMP modulating enzymes. For most of them, it is not known how and under what conditions they are activated, nor are their downstream effectors known. New insights into how the activity of individual c-di-GMP modulating regulons is integrated to drive bacterial behaviour towards biofilm formation might become the basis for developing new strategies to combat problematic chronic infections.

In this study, we reanalyzed previously recorded extensive RNA sequencing data of 414 clinical *P. aeruginosa* isolates (Dötsch et al. 2015a, Hornischer et al. 2019). We demonstrate that the identification of a subset of clinical isolates that express three different sigma factors (RpoS, RpoN, and AlgU) at high levels and another subset of clinical isolates that express the sigma factors at low levels, allowed for the identification of genes that were differentially regulated between those two subgroups. Interestingly, the set of differentially regulated genes reflected the regulon of the sigma factors, as identified in a previous study (Schulz et al. 2015).

We then tested, whether this approach could be of use to identify genes involved in c-di-GMP signaling pathways in the opportunistic pathogen *P. aeruginosa*. We concentrated our analysis on two DGCs encoded by PA14_23130 (PA3177) and PA14_03790 (PA0290). The transcription of both genes was positively correlated with the transcription of the gene cluster, which is involved in alginate biosynthesis. Alginate is a component of the protective biofilm matrix. In addition to the *pel* genes, the *alg* genes have been shown to be regulated by elevated c-di-GMP levels, and the *pel* and *alg* genes have been associated with increased biofilm formation (Hentzer et al. 2001, Valentini and Filloux 2016, Lee et al. 2007, Merighi et al. 2007, Whitney et al. 2012, 2015, Liang et al. 2022).

## Material and methods

### Bacterial strains and growth conditions

The strains used in this study are listed in Table S1 (Supporting Information). If not stated otherwise, all strains were cultured at 37°C in lysogeny broth (LB) medium (10 g/l tryptone, 5 g/l yeast extract, and 7 g/l NaCl) with gentle shaking, on LB plates [supplemented with 1.5% (w/v) agar] or on sheep blood agar plates (Th. Geyer).

### Deletion mutant strain construction

In order to obtain *P. aeruginosa* deletion mutants, the protocol developed by Hmelo et al. (2015) was followed. Primers for two DNA fragments flanking the gene of interest were used for PCR amplification (Table S3, Supporting Information). These fragments were then SOEing PCR-fused and cloned into pDONRPEX18Gm vector with BP Clonase (Invitrogen). The allelic-exchange insert of both plasmids was verified by Sanger sequencing. Merodiploids with the allelic-exchange vector (Table S2, Supporting Information) were created by triparental mating of the DH5α donor with an HB101/pRK600 helper, and the *P. aeruginosa* strain of interest (recipient). Subsequently, trans-conjugants were subjected to SacB-based counter selection on LB plates with 60 mg/ml gentamicin and no-NaCl-LB plates with 15% sucrose. Double cross-over trans-conjugants were identified via PCR analysis and Sanger sequencing. In this study, deletions of the gene *PA14_23130 (PA3177)* and *PA14_03790 (PA0290)* were constructed in the type strain PAO1 and clinical isolate CH3484.

### RNA extraction, RNASeq, and transcriptomic analysis

For RNA extraction, an overnight LB culture was diluted to an OD_600_ of 0.05 in 10 ml LB medium and grown while shaking (180 rpm) to early stationary phase (OD_600_ = 2). RNA was then extracted using the RNeasy Mini Kit (Qiagen) in combination with Qiashredder columns (Qiagen) according to the manufacturer’s instructions. Quality of the RNA was checked with the RNA Nano Kit (Agilent Technologies) on a fragment-analyzer (Agilent Bioanalyzer 2100, Agilent Technologies). Depletion of rRNA was done with the RiboZero Bacteria Kit (Illumina) and cDNA libraries were generated with the ScriptSeq v2 Kit (Illumina). For sequencing the RNA samples were run on an Illumina HiSeq 2500 device in single-end mode with 50 cycles, resulting in ∼37 million raw reads per sample (mean). The reads were then mapped with a stampy pipeline (Lunter and Goodson 2011) to the PAO1 or PA14 genome, respectively as reference. After initial removal of genes with more than 5 counts per million and genes that were absent in at least 98% of the isolates in the dataset, differentially expressed genes were determined with the R package edgeR (v.3.20.1). Comparison between the strains was done on the basis of differential gene expression (TREAT; edgeR function glmTreat). Significance thresholds were usually set to |log2 fold-change| > 2 and FDR < 0.05, if not indicated otherwise.

### Isolate gene-expression based gene-regulon calculation

Regulons based on gene expression of isolates were calculated for each gene individually. Isolates that did not show expression of the respective gene were excluded. Furthermore, only the top 4% of isolates with the highest and lowest expression were selected for differential expression analyses. Exceptions were made if more than five isolates were significant outliers with higher or lower expression. In this manner, the regulon was calculated between groups of 5–16 isolates (algorithm-based isolate selection described in Tables S4 and S5, Supporting Information). Outliers were calculated using the grDevices R package and the boxplot.stats function with default settings. After calculating differential expression (edgeR with glmTreat), the resulting lists of differentially expressed genes that had a |log2 fold-change| > 2 and a *P*-value < .05 were selected. When a set of differentially expressed genes reached a certain size, the cut-off was tightened to focus on more significant and specific results: If more than 10 hits were obtained with FDR < 0.1, the significance was set to FDR < 0.1. If more than 20 hits were obtained for FDR < 0.05, the significance was set to FDR < 0.05. If more than 200 hits for FDR < 0.05 and or more than 50 hits for FDR < 0.01 were found, the significance was set to FDR < 0.01. The applied thresholds for each gene regulon are highlighted in red in Tables S4 and S5 (Supporting Information).

### 96-well plate based growth assay

Growth behavior was monitored in a 96-well plate and OD was monitored in a BioTek Synergy plate reader at 37°C with double-orbital shaking at 537 rpm for a total of 18 h and an OD_600_ measurement interval of 20 min. Precultures were grown in 3 ml LB under standard conditions and diluted to an OD_600_ of 0.05, before 200 µl was transferred into the wells of the 96-well plate. The outer wells were filled with sterile water and each isolate was tested in three technical replicate wells, which were randomly distributed on the plate. In total, three individual experiments (biological replicates) were carried out.

### Swimming motility assay

Swimming motility assays were conducted following the protocol published by Ha et al. (2014a). Briefly, plates containing 0.3% swim agar were allowed to dry at room temperature (RT) for ∼4 h prior to inoculation. All swim plates were incubated upright at 37°C for 16–18 h prior to analyses using ImageJ software (NIH). Since *P. aeruginosa* motility can be visualized as a sharp ring of growth that forms as the bacteria move outward from the original inoculation point, swim zones of a strain were determined as the colony diameter in cm and averaged from three technical replicates per plate. As a negative control, PA14 Δ*fliC* with a known swimming deficiency was used.

### Swarming motility assay

Swarming motility assays were performed as previously described (Ha et al. 2014b). Briefly, freshly prepared swarm agar (0.5%) plates were left to dry at RT for approximately 1 h prior to inoculation. All swarm agar plates were incubated upright at 37°C for 16–18 h prior to analysis using ImageJ software (NIH). Since *P. aeruginosa* movement results the formation of tendrils or fractals radiating from the inoculation center, swarm zones of a strain were determined as the colony area in cm^2^ and averaged from two technical replicates per plate. As a negative control, PA14 Δ*fliC* with a known swarming deficiency was used.

### Twitching motility assay

Twitching motility assays were performed as previously described (Turnbull and Whitchurch 2014). Briefly, LB agar (1.5%) plates that were prepared the day before use were left to adjust to RT for approximately 1 h prior to stabbing holes into the agar with sterile shortened yellow tips. All twitching agar plates were incubated upright at 37°C for 24 h prior to removing the agar and staining the twitching area with 0.1% crystal violet (Sigma). The twitching area was analyzed using ImageJ software (NIH) and defined as the colony area in cm^2^. Average twitching areas from three technical replicates per plate were determined. As a negative control, either a PA14-like or PAO1-like clinical isolate with known twitch-deficiency was used (BACTOME).

### Biofilm quantification by crystal violet attachment assay

A crystal violet attachment assay was utilized to quantify the biofilms, with slight modifications from the method described by O’Toole (2011) including some minor modification. Overnight cultures were adjusted to an OD_600_ of 0.02 and 100 µl of the inoculum were aliquoted to one row of eight wells of a nontreated U-bottom PVC 96-well plate (Corning Inc., NY, USA). For incubation at 37°C, plates were sealed with an air-permeable membrane and placed in a humid incubator for 24 h or 48 h, respectively. The supernatant was removed and the wells were washed with water before adding 150 µl of a 0.1% w/v crystal violet staining. After 30 min of incubation, the staining solution was removed and the wells were washed and air-dried. 200 µl of 96% ethanol was added to each well for destaining and incubated for 30 min at RT. A volume of 125 µl of the solution was transferred to a fresh 96-well flat bottom plate and the absorbance was measured at 540 nm. Each strain was tested in two independent experiments with eight technical replicates each time.

### Statistical analysis

An analysis of variance was performed to reveal significant differences in values between each isolate and their respective wild-types using a pairwise two-tailed Student *t*-test in the GraphPad Prism software (GraphPad, La Jolla, CA).

## Results

### Impact of the expression level of global regulators on the overall transcriptional profiles of clinical isolates

**Figure 1.**
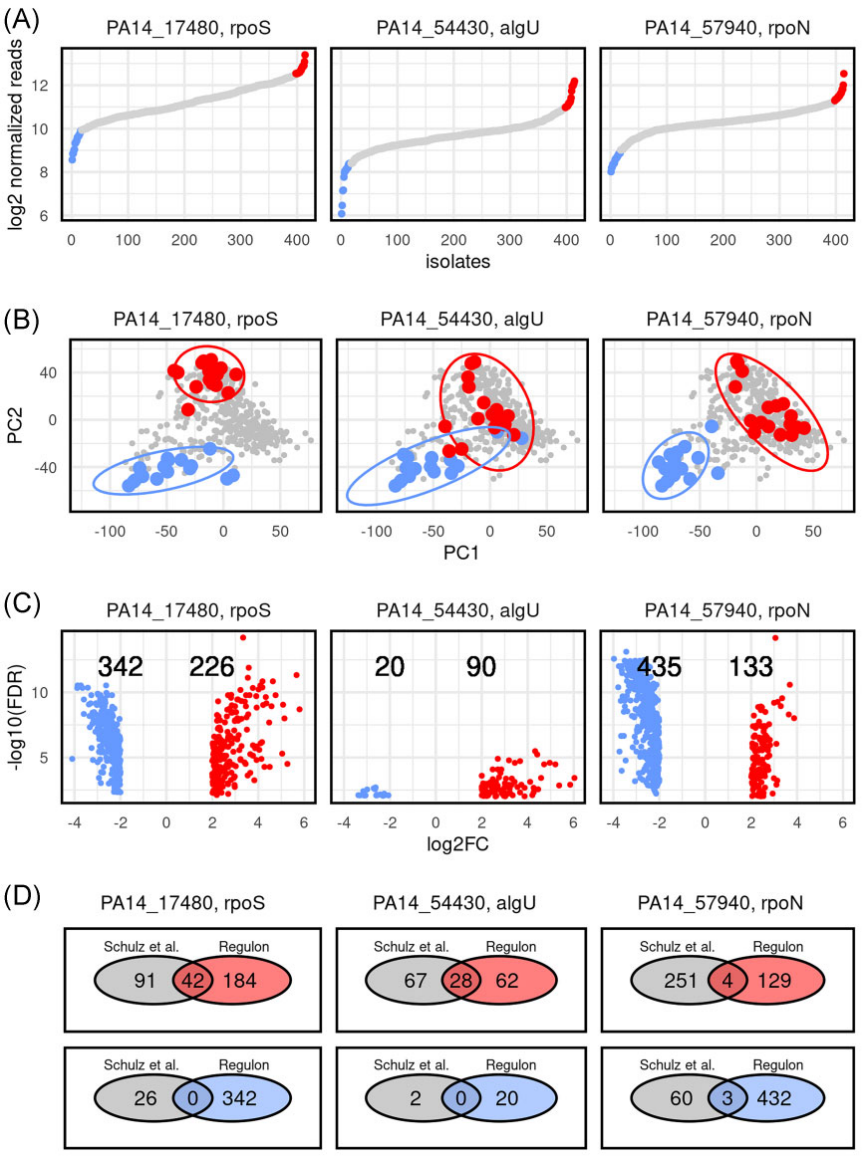
Sigma factor regulons in *P. aeruginosa* clinical isolates. **(A)** Normalized RNAseq reads of 414 clinical isolates (*x*-axis) sorted according to their sigma factor (RpoS, RpoN, and AlgU) gene expression levels (*y*-axis). The 4% of the isolates (amounting to 15–16 isolates) that express the genes at the highest (red) or lowest levels (blue) are colour-coded. **(B)** Expression profiles of all genes present in 98% of isolates (softcore genome) were used for a PCA. The ellipses represent the 95% confidence interval for the clinical isolate groups exhibiting a high (red) versus low (blue) expression of the respective gene. **(C)** Differentially expressed genes (log2 fold-change > 2; *P*-value of at least < .01) between isolates with high versus low expression of the three respective sigma factor genes are depicted. Numbers indicate the number of differentially expressed genes, which are either upregulated in high-expressing (red) or low-expressing isolates (blue). **(D)** Comparison of the genes that were differentially regulated between groups of clinical isolates expressing the respective sigma factor genes at high versus low levels (C) with genes that were previously described to belong to the respective sigma factor regulon (Schulz et al. 2015).

We have previously recorded transcriptional profiles of 414 *P. aeruginosa* clinical isolates grown under standard laboratory conditions (Dötsch et al. 2015a, Hornischer et al. 2019). Analyzing gene expression levels of individual genes across a multitude of clinical isolates allows for the selection of those clinical isolates that express any gene of interest at a high and low level, respectively. As shown in Fig. 1(A), we exemplarily selected clinical isolates with the highest expression level of the genes encoding for the three sigma factors RpoS, RpoN, and AlgU. The respective groups of high- and low- expressing isolates clustered separately in a principal component analysis (PCA) based on the expression of genes present in the majority of the clinical isolates (Fig. 1B). Thus, despite the fact that the various clinical isolates do not share the same genetic background, high or low expression of global regulators exhibited a common impact on the transcription of a large set of genes, so that the transcriptional profiles clustered in two distinct groups.

### Using the diversity of transcriptional profiles of clinical isolates to identify gene regulons

Since we found that differential expression of genes encoding global regulators affects the overall transcriptional profile of clinical isolates, we wanted to determine whether the RNA-seq data could be used to identify the regulons of global regulators, i.e. the genes that are differentially regulated in the presence or absence of the respective regulator. As depicted in Fig. 1(C), we found 568 genes that were differentially expressed between the two groups of clinical isolates that expressed *rpoS* at high versus low levels. The same number of genes were found to be differentially regulated between clinical isolates expressing *rpoN* at high versus low levels, whereas only 110 genes were found to be significantly different in the *algU* high versus low expressing clinical isolates. A total of 26.4% (42 genes) of the genes that were previously found to be differentially expressed in an *rpoS* deletion mutant as compared to its respective wild-type grown under *rpoS* inducing environmental conditions were also found to be differentially expressed in the *rpoS* high versus low expressing clinical isolates (Schulz et al. 2015) (Fig. 1D; and Table S5, Supporting Information). Similarly, 28.9% (28 genes) of the genes that were previously found to be differentially expressed in an *algU* deletion mutant as compared to its respective wild-type grown under *algU* inducing environmental conditions were found to be differentially expressed in the *algU* high versus low expressing clinical isolates. However, no big overlap was found for the RpoN regulon (2.2%, seven genes), despite the finding that the transcriptomes of the high versus low *rpoN* expressing clinical isolates were distinct from another. In conclusion, our data indicate that analyzing clinical isolates, which exhibit altered expression of global regulators under standard laboratory growth conditions, might provide a unique opportunity to overcome the challenge that for many c-di-GMP modulating enzymes it is not known under which conditions their expression is activated.

### Identification of clinical isolates exhibiting high versus low expression of c-di-GMP modulating enzymes

**Figure 2.**
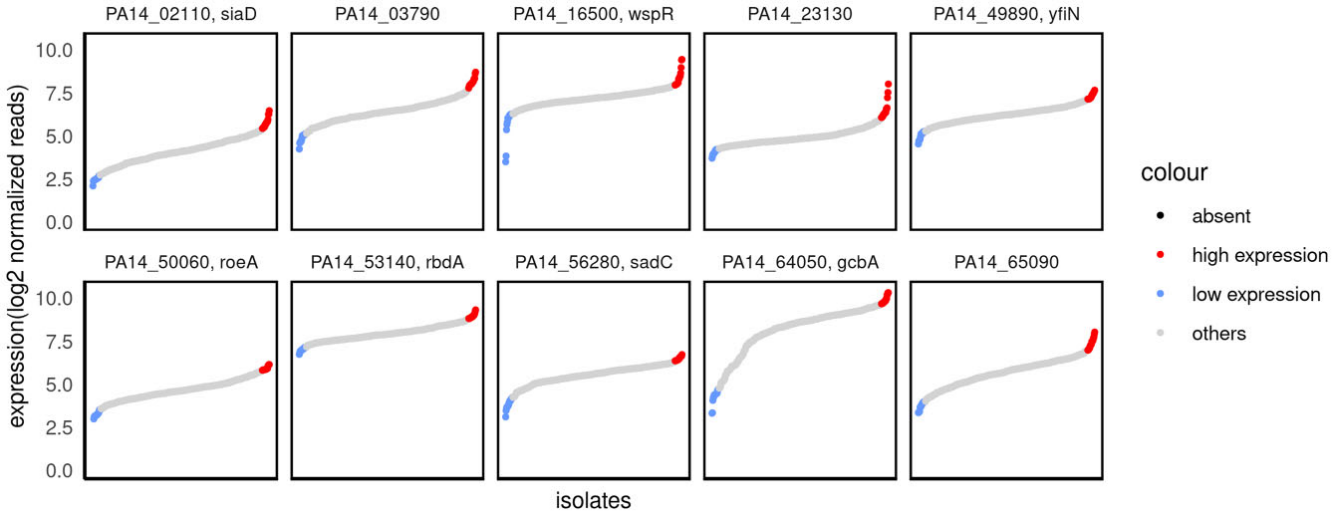
Gene expression levels across clinical isolates. Normalized RNAseq reads of 414 clinical isolates sorted along the *x*-axis according to the expression levels (*y*-axis) of genes encoding c-di-GMP modulating enzymes. The 4% of the isolates (amounting to 15–16 isolates) that express the genes at the highest (red) or lowest levels (blue) are colour-coded.

We selected clinical isolates that exhibited a high versus a low expression of genes encoding for c-di-GMP modulating enzymes under standard LB growth conditions. The higher transcriptional activity of genes encoding c-di-GMP modulating enzymes despite growth under noninducing conditions may allow uncovering details on the regulon of the respective c-di-GMP modulating enzyme. Figure 2 shows the divergence of the gene expression levels of a selection of 10 c-di-GMP modulating enzymes in the 414 clinical isolates (profiles for all c-di-GMP modulating enzymes can be found in Figure S1, Supporting Information). Most of the clinical isolates express the genes at a comparable level. However, the dataset on 414 clinical isolates seems large enough to also identify at least a few isolates that exhibit gene expression levels that are clearly different from the median for most of the genes encoding c-di-GMP modulating enzymes.

### Identification of the PA14_23130 and PA14_03790 c-di-GMP cyclase regulons

**Figure 3.**
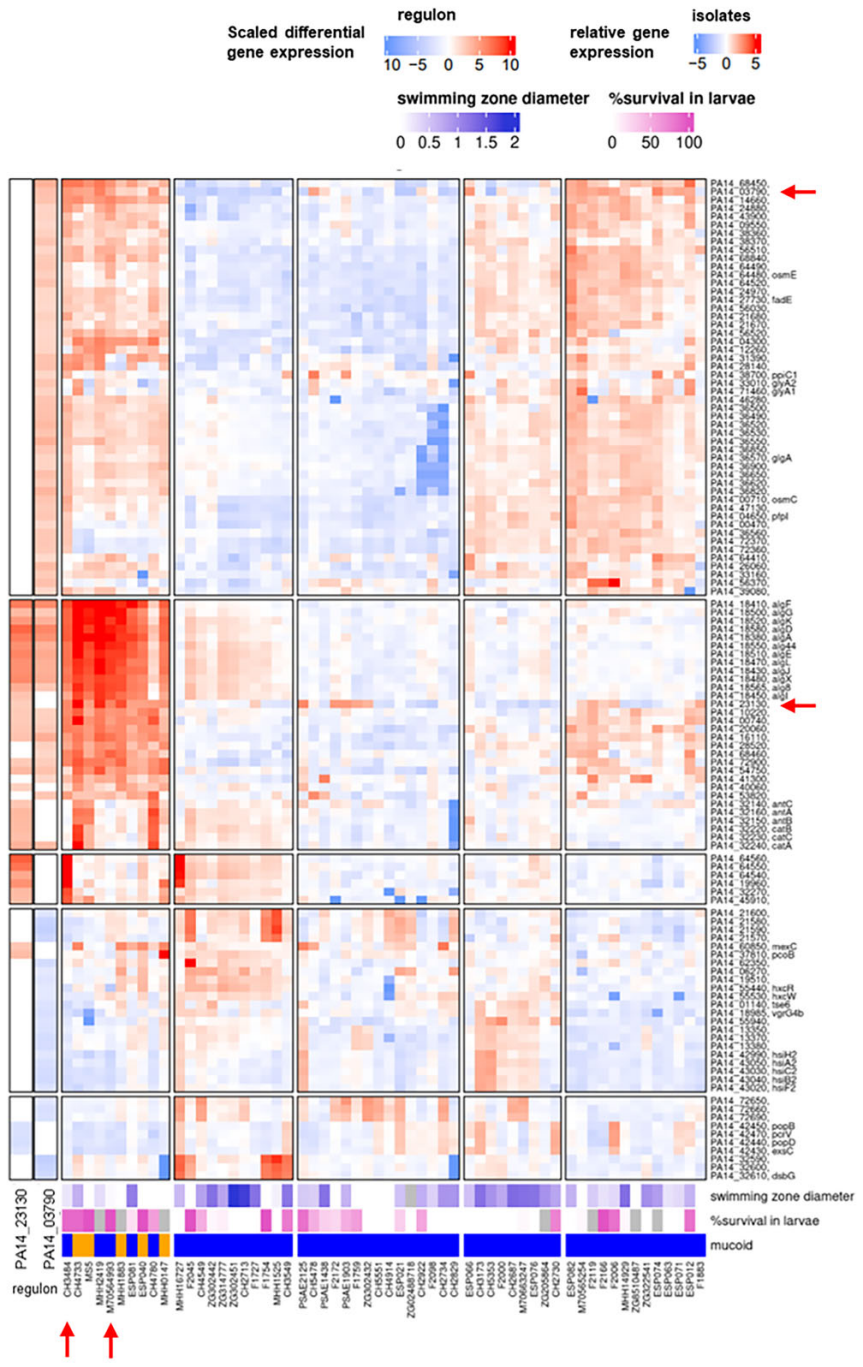
Alginate biosynthesis and PA14_23130 and PA14_03790 gene expression is coregulated in *P. aeruginosa* clinical isolates. The heat map depicts the expression level of all genes (*y*-axis) that were shown to be differentially expressed in those 58 clinical isolates (*x*-axis) that express either PA14_23130 or PA14_03790 at high or low levels (the respective regulons are indicated in the two vertical columns on the far left). High alginate gene expression correlates positively with expression of the GGDEF domain containing cyclases PA14_23130 and PA14_03790 (indicated by red arrows on the right side of the heatmap), negatively with swimming and positively with survival in *G. mellonella* larvae and a mucoid colony surface (orange = mucoid, blue = nonmucoid) (Hornischer et al. 2019). The clinical isolates CH3484 and M70564993 that were further analyzed in this study are marked by red arrows at the bottom of the heatmap.

We concentrated on the differentially expressed genes in groups of clinical isolates that expressed two genes encoding c-di-GMP cyclases (PA14_23130 and PA14_03790) at high versus low levels. A total of 38 genes were differentially regulated in groups of clinical isolates that expressed PA14_23130 at high versus low levels, whereas 98 genes were differentially regulated in groups of clinical isolates that expressed PA14_03790 at high versus low levels (Table S4, Supporting Information). As shown in Fig. 3, the expression of both cyclase genes was positively correlated to the expression of the alginate biosynthesis genes across the transcriptional profiles of the 414 clinical isolates. A positive correlation of the expression of these two cyclases and the alginate biosynthesis genes was also found in previous RNA-seq studies of *P. aeruginosa* isolates grown in artificial sputum medium (Huse et al. 2010, Damron et al. 2012).

**Figure 4.**
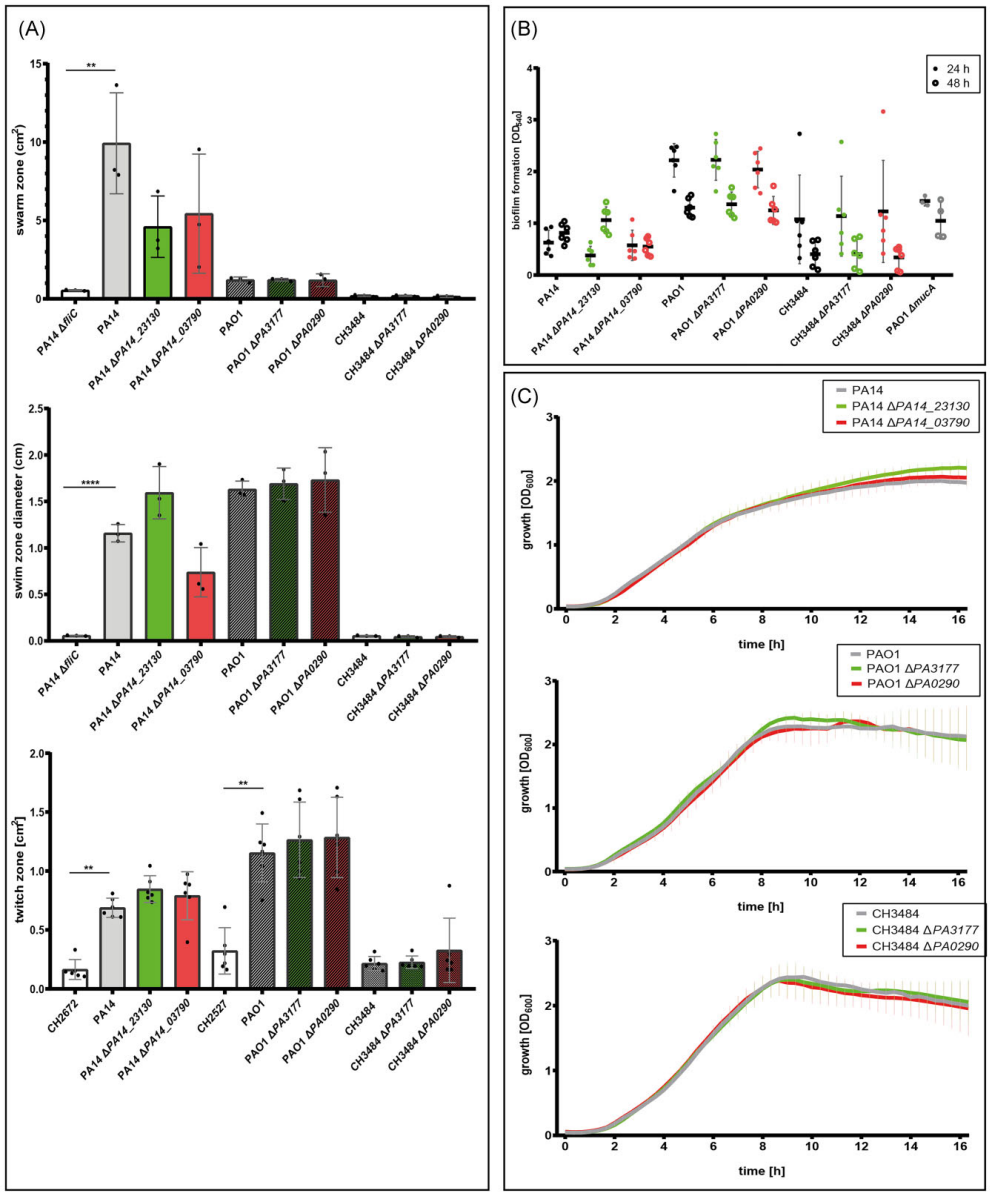
Deletion of PA14_23130 and PA14_03790 encoding for DGCs does not affect c-di-GMP regulated phenotypes. Motility phenotypes including swimming, swarming, and twitching were not significantly different in the PA14 ΔPA14_23130 and PA14 ΔPA14_03790, the corresponding PAO1 ΔPA3177 and PAO1 ΔPA0290 or the clinical isolate CH3484 ΔPA3177 and CH3484 ΔPA0290 deletion mutants compared to their respective wild-types. All experiments were performed in three replicates. A *fliC* deletion mutant served as a negative control **(A)**. The capability to form biofilm was measured using the crystal violet assay and was also not significantly different in the deletion mutants compared to their respective wild-types both after 24 (filled circles) and 48 h (empty circles). All experiments were performed in three replicates. A *mucA* (hypermucoid) deletion mutant served as control **(B)**. Growth was monitored in a 96-well plate in rich medium at 37°C in biological triplicates in three independent experiments. No differences in growth, motility, and biofilm formation were detected between the deletion mutants compared to their respective wild-types **(C)**. ^****^, *P* < .0005; **, *P* < .005.

In order to study the downstream effects of the PA14_23130 and PA14_03790 cyclase activity further, we deleted the two genes in the two *P. aeruginosa* type strains PAO1 and PA14 and recorded their motility phenotypes, growth behaviour and ability to produce biofilms. As depicted in Fig. 4, swimming, swarming and twitching motility was not altered in the cyclase gene mutants as compared to their respective wild-types. Furthermore, no difference in growth under standard rich medium conditions was observed and inactivation of the PA14_23130 (PA3177) or PA14_03790 (PA0290) gene did not impact biofilm formation in the PAO1 or PA14 strain background. We also recorded the transcriptional profile of the respective deletion mutants. As compared to their PA14 and PAO1 wild-types, 148 genes were differentially regulated in the PA14_23130 deletion mutant and 13 genes in the corresponding PAO1PAO1 PA3177 deletion mutant (Table S6, Supporting Information). The PA14_03790 deletion mutant exhibited two differentially regulated genes in the PA14 strain background and the corresponding PA0290 deletion mutant 25 genes in the PAO1 strain background. There were no genes that were differentially regulated in both deletion mutant strain backgrounds.

We hypothesized that inactivation of the two genes encoding the DGCs in a strain expressing the corresponding genes at elevated levels might provide more insight into the downstream regulon. We therefore generated CH3484 ΔPA3177 and CH3484 ΔPA0290 deletion mutants in the PAO1-like clinical isolate CH3483, which expressed both cyclase encoding genes at high levels. Again, inactivation of the two cyclase encoding genes did not have an impact on swimming, twitching and swarming activity. Furthermore, growth was not affected nor was biofilm formation (Fig. 4). We also recorded the transcriptional profile of the cyclase deletion mutants. We found 30 and 8 differentially regulated genes in the CH3484 ΔPA3177 and CH3484 ΔPA0290 deletion mutants as opposed to their corresponding wild-type, respectively. Of note, the transcriptional profiles of the CH3484 ΔPA3177 and CH3484 ΔPA0290 deletion mutants were almost identical and no gene was differentially regulated at a significant level between the two deletion mutants. We also found no gene that was differentially regulated in more than one ΔPA0290/ΔPA14_03790 deletion mutant strain background (PAO1, PA14 or CH3484) and only two genes (PA4888 and PA4889) that were differentially regulated in the ΔPA3177/Δ PA14_23130 deletion mutants in both the PAO1 and the CH4484 clinical strain background.

**Figure 5.**
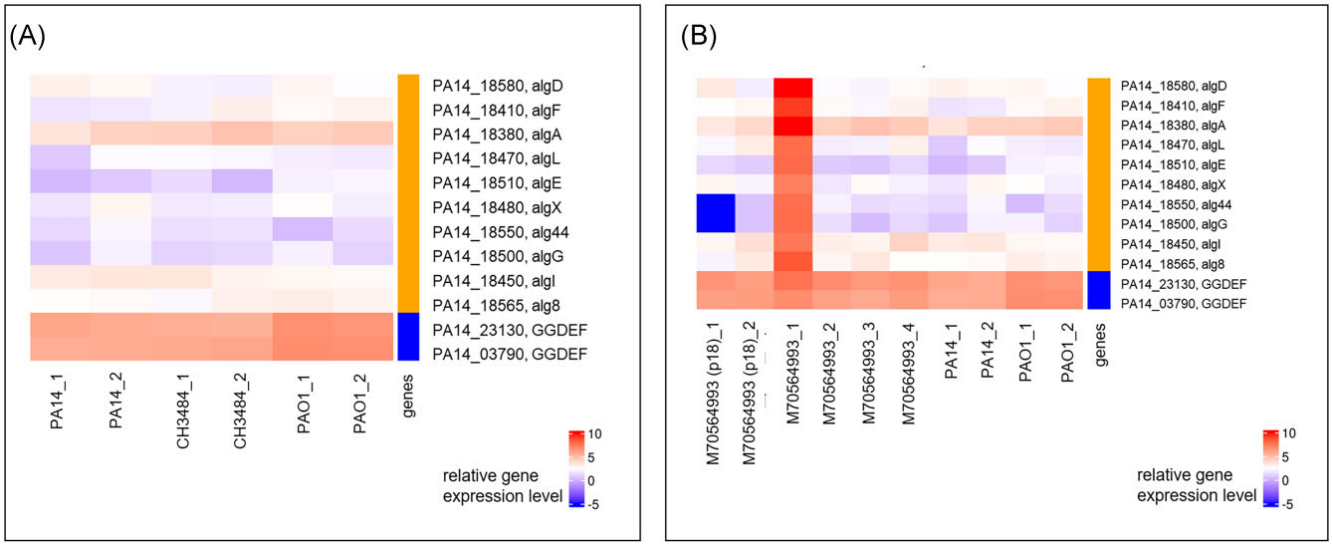
Log2 normalized read counts of the alginate biosynthesis genes and the GGDEF-domain containing enzymes PA14_23130 and PA14_03790. **(A)** Comparison of the respective gene expression levels in the clinical isolate CH3484 and the two type strains PA14 and PAO1 measured in duplicates. **(B)** Comparison of the respective gene expression levels in the clinical isolate M70564993 in four replicates (indicated by _1, _2, _3, and _4) and the M70564993 isolate after 18 passages (p18) in LB-medium.

Since we did not detect any gene involved in the production of alginate to be differentially regulated in either of the two cyclase mutants, we manually looked into the alginate biosynthesis gene cluster. In both CH3484 ΔPA3177 and CH3484 ΔPA0290 deletion mutants, the alginate biosynthesis genes were not expressed at a high level. However, a rerecording of the RNA-seq profile of the clinical isolate CH3484 revealed that the alginate gene cluster was also not expressed at an elevated level in CH3484. Instead and as opposed to previously recorded RNA-seq profile (Dötsch et al. 2015b, Hornischer et al. 2019; Fig. 3), the alginate gene cluster expression level of CH3484 compared well to that of the PA14 and PAO1 strains, indicating that the clinical isolate has lost its high alginate gene expression level (Fig. 5A).

We, therefore, selected a second clinical isolate (M70564993), which had been demonstrated to express the alginate genes at elevated levels (Fig. 3), and passaged the strain for overall 18 days by transferring 100 µl culture to 5 ml fresh LB medium twice per day. Figure 5(B) shows that while one replicate of a freshly thawed sample of M70564993 still expressed the alginate genes at elevated levels, in three other freshly thawed samples (and both of the passaged isolate samples) the high alginate gene expression levels were lost and comparable to that of the PA14 and PAO1 type strains.

## Discussion

Rising c-di-GMP levels drive the switch towards biofilm phenotypes. However, in the opportunistic pathogen *P. aeruginosa* there are various c-di-GMP modulating enzymes, which are responsible for different and in parts nonoverlapping bacterial phenotypes (Kulesekara et al. 2006, Lee et al. 2006, Römling et al. 2013, Ha et al. 2014c, Valentini and Filloux 2016). Only knowledge on the activity of the individual c-di-GMP signaling pathways, their regulons and how their activities are integrated to adjust bacterial behaviour will provide the basis to understand bacterial adaptation to complex environmental challenges, such as those found during an acute versus chronic infection process. In this study, we aimed at making use of available data on the transcriptional profiles of a plethora of clinical *P. aeruginosa* isolates (Dötsch et al. 2015a, Hornischer et al. 2019). We selected clinical isolates that express genes encoding for distinct c-di-GMP modulating enzymes at high levels even under conditions that normally would not induce the system. We expected that deletion of the respective c-di-GMP modulating enzymes in these clinical isolates could provide insight into the role of the enzymes and the regulated pathways, even if the environmental conditions that trigger the system are unknown. We could show that this approach worked well for the identification of the regulon of two out of three major sigma factors (AlgU, RpoS).

In this study, we concentrated on two not well characterized c-di-GMP cyclases (Poudyal and Sauer 2018, Wei et al. 2019, Bhasme et al. 2020), whose expression was found to be coregulated with genes involved in alginate biosynthesis in many of our clinical isolates, as well as under environmental conditions that mimic the cystic fibrosis lung environment (Alvarez-Ortega and Harwood 2007, Son et al. 2007, Tralau et al. 2007, Anderson et al. 2008, Fung et al. 2010, Bobadilla Fazzini et al. 2013). In order to unravel whether there is a casual relation between the high expression of the cyclases and the expression of alginate biosynthesis genes, we sought to inactivate the respective cyclases in the high cyclase expressing clinical isolate (CH3484) and characterize the downstream effects (Liang et al. 2022). As expected, the inactivation of the two cyclases did not elicit any motility, growth, biofilm, or alginate gene expression phenotype in the two *P. aeruginosa* type strains PA14 and PAO1, which do not express the two genes at elevated levels under standard laboratory growth conditions. However, we also did not detect a correlation between the expression of either cyclase or a motility, growth, biofilm, or alginate gene expression phenotype in the clinical isolate CH3484. Nevertheless, our results do not exclude a causal relationship either, because we found that the increased alginate gene expression was quickly lost in restreaked isolates of the CH3484 wild-type. The same observation was made for another clinical isolate, which following restreaking and passaging also lost the high expression of the *alg* genes. It is thus very likely that while constructing the corresponding cyclase gene deletion mutations in the clinical isolate the unstable phenotype of high *alg* gene expression was lost independently of the deletion of the cyclase genes.

Although our overall approach was not successful, our finding that high gene expression levels of the two cyclases, at least in the two strains analyzed in this study (CH3484 and M70564993) are not stable is interesting. These results fit well with a recent study published by us, where we show that elevated c-di-GMP levels in a clinical strain is gradually lost upon sub culturing (Koska et al. 2022). Interestingly, the gradual decline in elevated c-di-GMP concentrations appeared to be independent of genetic adaptation, but was reminiscent of a memory response in which the switch to low c-di-GMP concentrations in the studied *P. aeruginosa* isolate was delayed when the bacteria were cultured under non c-di-GMP-inducing conditions (Kordes et al. 2019).

**Figure 6.**
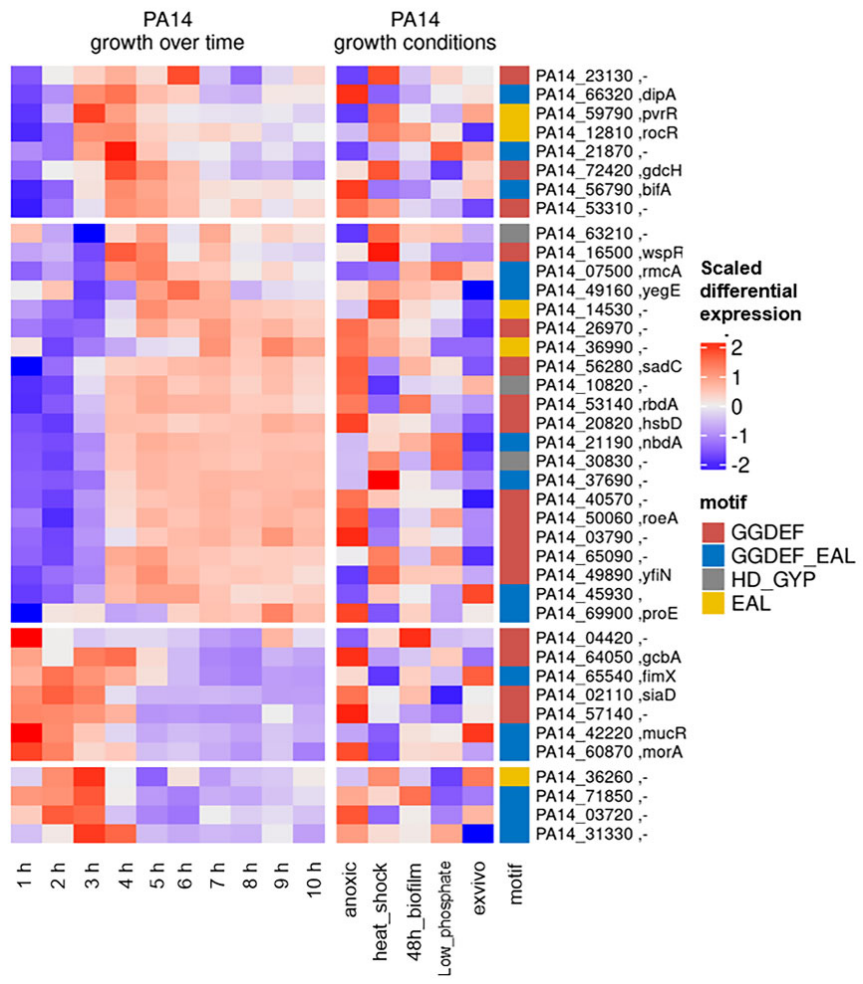
A systematic survey of the expression of the various *P. aeruginosa* c-di-GMP modulating enzymes under different environmental and growth conditions. On the left-hand panel relative expression of c-di-GMP regulating enzymes in a dataset were type-strain PA14 was grown in LB-medium for 10 h is shown (Alpers et al. 2023). The left-hand panel shows relative expression of the c-di-GMP regulating enzymes in PA14 under different growth conditions (Dötsch et al. 2015b) [The conditions are anoxic cultivation (anoxic), heat shock for 5 min at 50°C (heat shock), 48 h static biofilm growth (biofilm), minimal phosphate concentration (low phosphate), and expression in a mouse tumor infection model (ex vivo)]. Log2 normalized read counts were normalized per gene and dataset (z-score).

In conclusion, it appears that in the future one cannot avoid to construct strains that overexpress the cyclase of interest or even better, to identify the environmental conditions under which cyclase gene expression is activated and then determining the downstream effects in the mutant versus wild type under these conditions. A systematic survey of the expression of the various *P. aeruginosa* c-di-GMP modulating enzymes under different environmental conditions as depicted in Fig. 6 could be a good starting point.

## Supplementary Material

xtad012_Supplemental_Files

## Data Availability

Transcriptional data used in this study have been previously uploaded to the Gene Expression Omnibus (GEO) database with the accession numbers GSE123544, GSE159698, and GSE217100. Transcriptional data of the deletion mutants that were recorded in this study were uploaded with the accession number GSE233207. The genomic data of the 414 clinical isolates were previously uploaded to the Sequence Read Archive (SRA) with the accession number PRJNA526797.
