# Supplementary material for: Challenges in using transcriptome data to study the c-di-GMP signaling network in Pseudomonas aeruginosa clinical isolates
Source: FEMS Microbes. 2023 Jul 18;4:xtad012. doi: 10.1093/femsmc/xtad012 (PMC10411656; doi:10.1093/femsmc/xtad012)
Supplement: xtad012_Supplemental_Files [file xtad012_supplemental_files.zip › Supplements_Figures.docx]

**Supplements**

**Table S1** Strains used in the study.

| Strain | Relevant genotype | Reference/source |
| --- | --- | --- |
| *P. aeruginosa* strains |  |  |
| M70564993 | PA14-like clinical isolate of *P. aeruginosa* | Bactome collection (Hornischer et al., 2019) |
| CH3484 | PAO1-like clinical isolate of *P. aeruginosa* | Bactome collection (Hornischer et al., 2019) |
| CH3484 Δ*PA3177* | CH3484 (Δ*PA3177)* | this study |
| CH3484 Δ*PA0290* | CH3484 (Δ*PA0290)* | this study |
| PAO1 | *P. aeruginosa* reference strain |  |
| PAO1 Δ*PA3177* | PAO1 (Δ*PA3177)* | this study |
| PAO1 Δ*PA0290* | PAO1 (Δ*PA0290)* | this study |
| PA14 | *P. aeruginosa* reference strain |  |
| PA14 Δ*PA14_23130* | PA14 (Δ*PA14_23130)* | (Ha, Richman and O’Toole 2014a) |
| PA14 Δ*PA14_03790* | PA14 (Δ*PA14_03790)* | (Ha, Richman and O’Toole 2014a) |
| SH102 | PA14 Δ*fliC* |  |
| SD4 | PAO1 Δ*mucA* (G430Δ); mucoid | (Damkiær et al., 2013) |
| CH2672 | PA14-like clinical isolate of P. aeruginosa (twitch control) | Bactome collection (Hornischer et al., 2019) |
| CH2527 | PAO1-like clinical isolate of P. aeruginosa (twitch control) | Bactome collection (Hornischer et al., 2019) |
| *E. coli* strains |  |  |
| DH5α | F ^-^ *endA1 glnV44 thi-1 recA1 relA1 gyrA96 deoR nupG purB20 Φ80dlacZΔM15 Δ(lacZYA-argF) U169 hsdR17 (r_k_ ^-^ m_k_ ^+^) λ^-^* |  |
| HB101 | *recA thi pro leu hsdRM1,* Sm^r^ | (Kessler et al., 1992) |
| Rosetta DE3 pLysS | F ^-^ *ompT hsdSB(r_B_ ^-^ m_B_ ^-^) gal dcm* DE3 pLysSRARE, Cm^r^ | Novagen |

**Table S2** Plasmids used in the study.

| Plasmid | Description | | | Reference |
| --- | --- | --- | --- | --- |
| pDONRPEX18Gm | | Gateway donor, Gm^r^ | (Hoang et al., 1998) | |
| pRK600 | | Mobilization plasmid, Cm^r^ | (Kessler et al., 1992) | |
|  | |  |  | |
| *Knock-out vectors* | |  |  | |
| p*ΔPA3177* | | pDONRPEX18Gm based *ΔPA3177* knockout vector, Gm^r^ | This study | |
| p*ΔPA0290* | | pDONRPEX18Gm based *ΔPA0290* knockout vector, Gm^r^ | This study | |

**Table S3** Oligonucleotides used in the study.

| Oligo name | Nucleotide sequence 5‘ 🡪 3‘ | Reference |
| --- | --- | --- |
|  |  |  |
| PA3177-up-F | GGGGACAAGTTTGTACAAAAAAGCAGGCTCAGCTACTGACGATCTTCGTGCTG | This study |
| PA3177-up-R | CGAGGCTGATCGACAGCTTCAGGGACTGCTTGGGAGGAGCCAT | This study |
| PA3177-down-F | CTGAAGCTGTCGATCAGCCTCG | This study |
| PA3177-down-R | GGGGACCACTTTGTACAAGAAAGCTGGGTAAGGGCGAGGTGATCGACAAG | This study |
| PA3177-seq-F | ATCGTCCTGCGCAACCTCGA | This study |
| PA3177-seq-R | ATGCACAGGATCCTGGCTATCG | This study |
| PA0290-up-F | GGGGACAAGTTTGTACAAAAAAGCAGGCTCA GGCTACATGGCGGAAAACCA | This study |
| PA0290-up-R | TCAGCCCACGACGATGCAGTCAATGCCGGTTAGGTCGTCCAT | This study |
| PA0290-down-F | GACTGCATCGTCGTGGGCTGA | This study |
| PA0290-down-R | GGGGACCACTTTGTACAAGAAAGCTGGGTAAACCCGCGACGATGGAACGA | This study |
| PA0290-seq-F | CTGCTCAAGCTGCACAC | This study |
| PA0290-seq-R | TGCTAATTGTCTTCCGGTC | This study |

**Table S4 Regulon of all 40 c-di-GMP modifying enzymes present in the *P. aeruginosa* type strain PA14 based on previously recorded RNA-seq data of 414 clinical isolates.** Genes, whose expression level were shown to be differentially expressed (DEG) between groups of clinical isolates that express the various genes encoding the c-di-GMP modifying enzymes at high versus low levels, are listed. Cut-off for significance of DEG was set depending on the number of genes found and varied from a p-value of 0.05 as the least stringent and up to an FDR of 0.01 as the most stringent conditions. The cut-off chosen for the results illustrated in each tab is highlighted in red. Only genes are listed that were at least 4-fold differentially regulated between the groups of high versus low gene expression clinical isolates.

**Table S5** Regulon of RpoS, RpoN and AlgU

**Table S6** Differentially expressed genes in mutants Regulon of RpoS, RpoN and AlgU


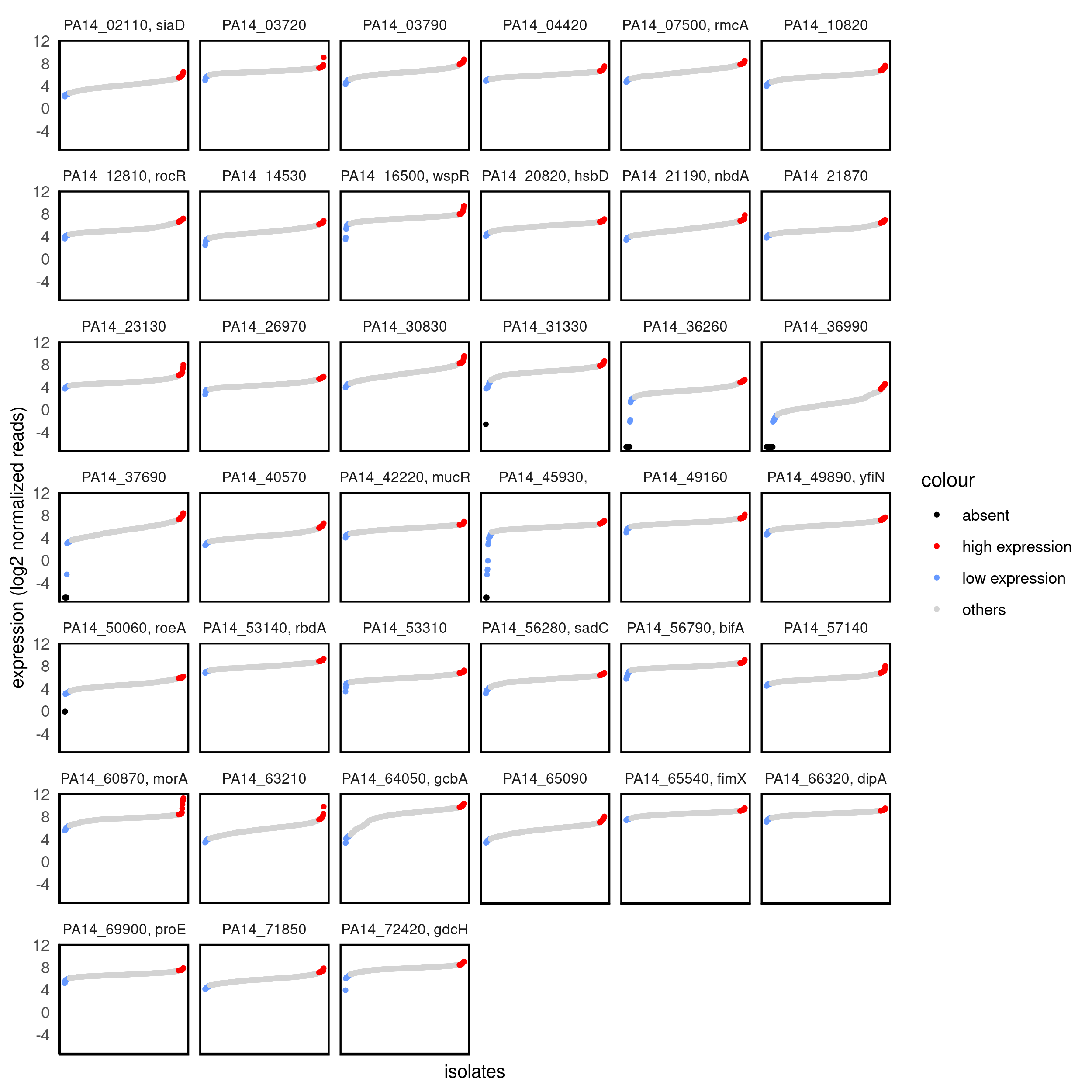


**Figure S1** Normalized RNAseq reads of 414 clinical isolates sorted along the x-axis according to the expression levels (y-axis) of 39 PA14 genes that encode c-di-GMP modulating enzymes - ordered according to their GGDEF/EAL/HD-GYP domain structure. (PvrR was excluded in this analysis, because of the absence of this gene in the clinical PAO1-like isolates). The 4 % of the isolates (accounting to approximately 15-16 isolates) that express the genes at the highest (red) or lowest levels (blue) are color-coded. Some genes were absent in the clinical isolates. This is indicated in black.

**References**

Damkiær, S., Yang, L., Molin, S., & Jelsbak, L. (2013). Evolutionary remodeling of global regulatory networks during long-term bacterial adaptation to human hosts. *Proceedings of the National Academy of Sciences of the United States of America*, *110*(19), 7766–7771. https://doi.org/10.1073/PNAS.1221466110/-/DCSUPPLEMENTAL/SM01.MOV

Ha, D. G., Richman, M. E., & O’Toolea, G. A. (2014). Deletion mutant library for investigation of functional outputs of cyclic diguanylate metabolism in *Pseudomonas aeruginosa* PA14. *Applied and Environmental Microbiology*, *80*(11), 3384–3393. https://doi.org/10.1128/AEM.00299-14

Hoang, T. T., Karkhoff-Schweizer, R. R., Kutchma, A. J., & Schweizer, H. P. (1998). A broad-host-range F1p-FRT recombination system for site-specific excision of chromosomally-located DNA sequences: Application for isolation of unmarked *Pseudomonas aeruginosa* mutants. *Gene*, *212*(1), 77–86. https://doi.org/10.1016/S0378-1119(98)00130-9

Hornischer, K., Khaledi, A., Pohl, S., Schniederjans, M., Pezoldt, L., Casilag, F., Muthukumarasamy, U., Bruchmann, S., Thöming, J., Kordes, A., & Häussler, S. (2019). BACTOME - a reference database to explore the sequence- and gene expression-variation landscape of *Pseudomonas aeruginosa* clinical isolates. *Nucleic Acids Research*, *47*(D1), D716–D720. https://doi.org/10.1093/nar/gky895

Kessler, B., de Lorenzo, V., & Timmis, K. N. (1992a). A general system to integrate *lacZ* fusions into the chromosomes of gram-negative eubacteria: regulation of the Pm promoter of the TOL plasmid studied with all controlling elements in monocopy. *Molecular & General Genetics : MGG*, *233*(1–2), 293–301. https://doi.org/10.1007/BF00587591
